# Should a low-protein diet and ketoanalogue supplementation be part of the management of advanced chronic kidney disease?

**DOI:** 10.1590/2175-8239-JBN-2024-0237en

**Published:** 2025-03-10

**Authors:** Yoko Narasaki, Hyung-Ah Jo, Connie M. Rhee

**Affiliations:** 1 University of California Los Angeles, Davd Geffen School of Medicine, Department of Medicine, Los Angeles, EUA.; 2 Tibor Rubin Veterans Affairs Long Beach Healthcare System, Long Beach, EUA.; 3 Inje University Ilsan Paik Hospital, Department of Internal Medicine, Gyeonggi-do, Coreia.; 4 University of California Irvine, Division of Nephrology, Hypertension, and Kidney Transplantation, Orange, EUA.; 5 Veterans Affairs Greater Los Angeles Healthcare System, Nephrology Section, Los Angeles, EUA.

**Keywords:** Diet, Protein-Restricted, Ketoanalogue Supplementation, Renal Insufficiency, Chronic, Dieta com Restrição de Proteínas, Suplementação com Cetoanálogos, Insuficiência Renal Crônica

## Abstract

The vast majority of patients with advanced chronic kidney disease (CKD) who transition to end-stage kidney disease (ESKD) are treated with dialysis. Given that dialysis does not always have the intended effects of increasing longevity and/or improving health, particularly in those with high comorbidity burden and/or older age groups, there has been increasing emphasis on interventions that delay or avert the need for renal replacement therapy. Among the multi-disciplinary approaches used to reduce CKD progression, dietary interventions are a major cornerstone. Current guidelines support the role of a low-protein diet in patients with moderate to advanced CKD who are metabolically stable. In addition to dietary protein amount, there is evidence that dietary protein sources as well as nutrients in plant-based foods have an important impact on kidney health outcomes. Clinical practice guidelines, including the 2020 National Kidney Foundation and Academy of Nutrition and Dietetics Kidney Disease Outcomes Quality Initiative Clinical Practice Guidelines for Nutrition in CKD, recommend a low protein diet for patients with moderate to advanced non-dialysis dependent (NDD)-CKD who are metabolically stable to reduce risk of ESKD and death. In addition to recommending lower protein intake, the recent 2024 Kidney Disease Improving Global Outcomes CKD Guidelines include a Practice Point advising that people with CKD eat more plant-based foods than animal-based foods. Increasing data also show that plant-based diets are associated with lower risk of progression of CKD and its complications including cardiovascular disease (cardio-kidney-metabolic syndrome), acid-base balance disorders, mineral bone disease, and dysglycemia.

## Resumo

A maioria dos pacientes com DRC avançada evoluindo para doença renal em estágio terminal (DRET) é tratada com diálise. Como a diálise nem sempre apresenta os efeitos pretendidos de aumentar longevidade e/ou melhorar a saúde, especialmente naqueles com alta carga de comorbidades e/ou faixas etárias mais avançadas, tem havido ênfase crescente em intervenções que retardem ou evitem a necessidade de TRS. Dentre abordagens multidisciplinares utilizadas para reduzir a progressão da DRC, intervenções dietéticas são um dos principais pilares. Diretrizes atuais sustentam o papel da dieta hipoproteica em pacientes com DRC moderada a avançada metabolicamente estáveis. Além da quantidade de proteína da dieta, existem evidências de que fontes de proteína dietética, bem como nutrientes dos alimentos de origem vegetal, têm impacto importante nos desfechos da saúde renal. As diretrizes de prática clínica, incluindo as Diretrizes de Prática Clínica para Nutrição na DRC da *National Kidney Foundation e da Academy of Nutrition and Dietetics Kidney Disease Outcomes Quality Initiative* de 2020, recomendam dieta de baixa proteína para pacientes com DRC não dependente de diálise (DRC-NDD) moderada a avançada, metabolicamente estáveis, para reduzir risco de DRET e óbito. Além de recomendar menor ingestão proteica, recentes Diretrizes de 2024 do KDIGO para DRC incluem um Ponto de Prática orientando pessoas com DRC a consumirem mais alimentos de origem vegetal do que animal. Dados crescentes também mostram que dietas à base de vegetais estão associadas a menor risco de progressão da DRC e suas compli­cações, incluindo doenças car­diovasculares (síndrome cardiovascular-renal-metabólica), distúrbios do equilíbrio ácido-base, distúrbio mineral ósseo e disglicemia.

## Introduction

**Figure 1:**
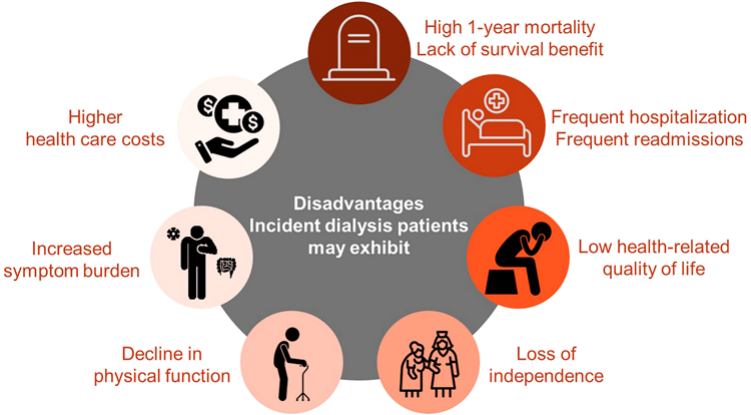
Challenges for certain subpopulations of advanced CKD patients transitioning to dialysis.

Each year, nearly 126,000 people in the United States (US) with chronic kidney disease (CKD) progress to end-stage kidney disease (ESKD), in which the kidneys reach an advanced loss of function and the patient requires renal replacement therapy (RRT) to survive^1^. While in-center hemodialysis has been the dominant treatment strategy across various options for RRT^1^, a growing number of studies have shown that dialysis does not always have the intended effects of increasing life or restoring health (Figure 1). A large body of evidence has shown that incident dialysis patients exhibit high rates of early mortality, and that there may be no survival benefit, frequent hospitalizations, lower health-related quality of life, loss of independence, decline in physical function, increased symptom burden, and higher health care costs particularly among certain subpopulations (i.e., high comorbidity burden, older age, etc.). For this reason, the US government launched the Advancing American Kidney Health Initiative in 2019, in which one of the key mandates is to encourage interventions that reduce the risk of incident CKD and CKD progression^2^.

**Figure 2:**
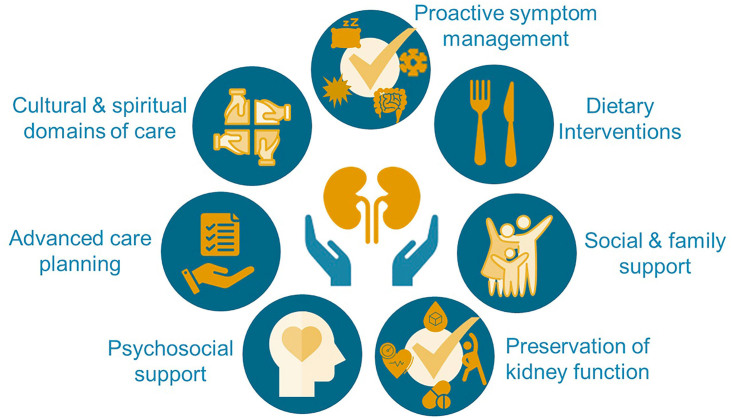
A multifaceted approach for conservative and preservative management of chronic kidney disease.

In the multi-faceted approach to reduce the risk of CKD and its progression, the importance of dietary interventions, and in particular, plant-dominant low-protein diets, in the primary and secondary prevention of CKD is increasingly recognized (Figure 2)^3^. In this perspective/opinion manuscript, we focus on dietary management, one of the major components of the multi-disciplinary approach to delay or avert the need for RRT. We specifically discuss the premise for low-protein diets (LPDs) and supplemented very low-protein diets (VLPDs) using essential amino acids or ketoacid analogs in the dietary management of advanced CKD patients. Our discussion also expands upon the role of plant-based diets and their practical implication in CKD patients.

## Discussion

### Rationale for Low Protein Diets in Chronic Kidney Disease

In the general population, high-protein diets have been increasingly utilized as a means to build muscle with resistance training^4^, maintain muscle mass with aging^5^, and promote weight loss during energy restriction particularly among those with obesity and diabetes^6,7,8^. High protein diets such as Atkins (i.e., 90% calories from protein and fat), Paleo (i.e., 19–35% calories from animal protein), South Beach (i.e., 70% calories from lean protein and unsaturated fat), ZONE (i.e., 30% calories from protein and 30% calories from fat), and KETO (i.e., 20–30% calories from protein and 60–80% calories from fat) are also popular in secular culture. Population-based data from a nationally representative cohort of US adults showed that dietary protein consumption exceeds the levels recommended by the Institute of Medicine for healthy adults and the National Kidney Foundation (NKF) and Academy of Nutrition and Dietetics Kidney Disease Outcomes Quality Initiative (KDOQI) guidelines for stages 3–5 non-dialysis dependent (NDD) CKD^9^.

Given the potential negative effects of high protein intake on kidney outcomes^10^, LPDs are a cornerstone of nutritional therapy in the conservative and preservative management of CKD^3^. Findings from animal models and clinical studies have shown that higher dietary protein intake causes increased glomerular filtration and intra-glomerular pressure, resulting from dilation of afferent arterioles and increased kidney blood flow to excrete protein-derived nitrogenous waste products^11,12,13^. Indeed, longitudinal studies in CKD patients have observed a linear relationship between lower protein intake and favorable CKD outcomes including reduced glomerular hyperfiltration, proteinuria, and CKD progression over time^14^. In addition to preventing and/or reducing the risk of CKD progression, LPDs may offer other metabolic benefits including better control of phosphorus homeostasis^15,16,17^, metabolic acidosis^18^, dyslipidemia, proteinuria, and inflammation^19,20,21^ as well as higher survival^22^. A meta-analysis of 16 randomized controlled trials of lower protein intake (<0.8 g/kg/day) vs. higher protein intake (≥0.8 g/kg/day) in NDD-CKD patients showed that LPDs conferred more favorable CKD outcomes including lower risk of ESKD, lower all-cause mortality, higher serum bicarbonate, and lower serum phosphorus levels; notably, there were no differences in nutritional indices across the two groups^23^. Consequently, the most recent NKF and Academy of Nutrition and Dietetics KDOQI nutrition guidelines recommend lower protein intake in metabolically stable non-diabetic CKD and diabetic CKD patients (0.55–0.6 g/kg/day and 0.6–0.8 g/kg/day, respectively)^24,25^, with a similar endorsement for metabolically stable individuals with NDD-CKD by the Kidney Disease Improving Global Outcomes (KDIGO) guidelines (0.8 g/kg/day)^26^.

Although the Modification of Diet in Renal Disease (MDRD) study showed that CKD progression is minimally attenuated by a LPD^27^, the trial had several key limitations (i.e., patients with diabetes treated with insulin were not included, the effects of short-term vs. long-term effects of the outcome on eGFR slope were not accounted for, relatively short follow-up time to detect differences in dietary groups^21,28^). The investigators then emphasized that the published primary results were inconclusive with respect to the efficacy of the dietary intervention, and they cautioned against interpreting the findings that LPDs do not reduce CKD progression as a “misinterpretation of inconclusive evidence as evidence in favor of the null hypothesis”^28^. The investigators also concluded that, although not definitive, based on multiple secondary analyses of the trial “the balance of evidence is more consistent with the hypothesis of a beneficial effect of protein restriction than with the contrary hypothesis of no beneficial effect”^28^.

### Plant-Based Diets in Chronic Kidney Disease

There has been rising interest in the role of plant-based diets in NDD-CKD given growing evidence of the benefits of both the amount and source of protein intake in this population^29^. The potential benefits of plant-based diets on kidney health outcomes include 1) attenuating risk factors for CKD (i.e., hypertension, diabetes and glycemia, obesity, and dyslipidemia), 2) lowering the risk of incident CKD and CKD progression, and 3) mitigating CKD-related complications (i.e., hypertension, cardiovascular disease, hyperphosphatemia, uremic toxins, inflammation, oxidative stress, metabolic acidosis, gastrointestinal disorders, and mortality)^29^. It is also plausible that the potential health benefits of plant-based diets may be due to their favorable nutrient profile, acid-neutral characteristics of plant-based proteins, and lower generation of uremic toxins and advanced glycation end products (AGEs) as compared to animal-based proteins^29,30^.

In general, vegan and vegetarian diets and any diet comprised of a larger proportion of foods from plant-based sources as opposed to animal-based sources can be categorized as plant-based diets^29^. In the nephrology field, examples of plant-based diets that have been proposed as a means to reduce the progression of kidney disease are 1) low protein vegan diets (0.7 g/kg/day of protein), 2) low protein supplemented vegan diets (0.6 g/kg/day of protein supplemented with essential amino acids and keto acids, i.e., one tablet per 10 kg of body weight), 3) VLPDs (0.3 g/kg/day of protein supplemented with essential amino acids and keto acids, i.e., one tablet for every 5 kg of body weight), 4) Plant-Dominant Low-Protein Diet (PLADO), and 5) Plant-Focused Nutrition in Patients With Diabetes and CKD Diet (PLAFOND)^29^.

### Role of Keto Acid Analogues in the Dietary Management of Chronic Kidney Disease

In adults with stages 3–5 NDD-CKD at risk of kidney failure, the KDIGO guidelines support consideration of a VLPD (0.3–0.4 g/kg/day) supplemented with essential amino acids or keto acid analogs (up to 0.6 g/kg/day) under close supervision^26^. Keto acids are paired with VLPDs in order to reduce nitrogenous wastes while maintaining adequate amino acid levels. Given the absence of an amino group in their chemical structure, keto acids can be used in place of their respective amino acids without providing nitrogen waste products while supporting nutritional adequacy^31^. Currently, keto acids are available as tablets or in powder form. One of the keto acid tablets used globally is Ketosteril^®^, which contains L-lysine (105 mg), L-threonine (53 mg), L-histidine (38 mg), L-tyrosine (30 mg), L-tryptophan (23 mg), hydroxy-methionine (59 mg), calcium-keto-valine (86 mg), calcium-keto-phenylalanine (68 mg), calcium-keto-leucine (101 mg), and calcium-ketoisoleucine (67 mg)^32^.

Limited data also show the safety and efficacy of supplemented VLPDs in clinically relevant subgroups. For example, a landmark randomized controlled trial showed the efficacy of supplemented VLPDs in elderly NDD-CKD patients (≥70 years old) in lowering the risk of CKD progression. A VLPD (defined as 0.3 g/kg/day of protein) supplemented with keto acid analogs, essential amino acids, and vitamins successfully delayed dialysis initiation by approximately 11 months compared to those without dietary protein intake restriction^33^. Data on the use of keto acids in CKD has also confirmed its safety and effectiveness^34,35^. For example, in a study of 81 patients with CKD and 116 patients with non-diabetic CKD, supplemented VLPDs were observed to be safe and have metabolic/nutritional benefits in both groups with respect to reduction of serum urea, phosphorus levels, and fasting glucose levels as well as nutritional markers including serum albumin, cholesterol, body weight, BMI, muscle strength^34^. In another study of stage 5 NDD-CKD patients with diabetes, the five-year mortality rate was 27% lower among keto acid users (N = 1001) vs. keto acid non-users (N = 14,781)^35^. This study also found that the incidence of ESKD and major adverse cardiovascular events among keto acid users was decreased by 35% and 23%, respectively, compared to keto acid non-users^35^.

### Practical Implementation of Low Protein Diets in Chronic Kidney Disease

The safety and efficacy of LPDs, including plant-based LPDs, are supported by a growing body of evidence. Data show that these diets have an adequate nutritional value and provide a sufficient quantity and quality of protein.

### Nutrition Adequacy

LPDs and plant-based diets as a means to reduce progression of CKD and its complications have sometimes been hindered by concerns about their nutritional adequacy and potential risk of protein energy wasting (PEW) in people with CKD. It worth noting that the development of PEW in CKD is not solely due to unintentionally low dietary intake, but is also caused by complex mechanisms including loss of appetite, higher levels of inflammatory cytokines, uremic toxins, and impaired gut microbiota, as well as altered conditions such as hypercatabolism, metabolic acidosis, and insulin resistance^36^. Indeed, LPDs have not been associated with the development of PEW in published studies. However, energy intake of participants in these studies is carefully monitored to ensure that it is adequate irrespective of the amount of protein intake^37^. Thus, in the practical implementation of LPDs, collaboration with trained kidney dietitians is recommended^38^ to ensure sufficient energy intake while maintaining protein intake within target ranges to maximize the effectiveness in reducing CKD progression while decreasing the risk of PEW^36^. There has been misconception that plant-based diets may require close monitoring to ensure adequate nutritional and caloric intake. However, a systematic review has shown that energy intake is comparable between individuals on a plant-based and animal-based diet^39^.

### Quantity and Quality of Protein

The adequacy of the quantity and quality of dietary protein in LPDs and plant-based diets has also been widely debated. However, it should be underscored that the thresholds for dietary protein intake for CKD patients are safe and nutritionally adequate based on metabolic studies, which have shown that a dietary protein intake of ~0.46 g/kg/day with essential amino acids avoids negative nitrogen balance. Hence, a protein intake of 0.6 g/kg/day (i.e. the lower threshold of recommended intake for stages 3–5 NDD-CKD) provides a 33% buffer to avoid falling below this threshold. It should also be highlighted that the recommended daily allowance (RDA) for protein intake in healthy adults is 0.8 g/kg/day. The estimated average dietary protein requirement for non-pregnant, non-lactating healthy adults is 0.6 g/kg/day. Thus, protein consumption of 0.8 g/kg/day provides an additional 33% safety buffer, which is endorsed by the Food and Agriculture Organization of the United Nations, World Health Organization, and Board of the National Academy of Sciences. Thus, a dietary protein intake of 0.8 g/kg/day (i.e. the upper threshold of recommended intake for stages 3–5 NDD-CKD) is similar to the protein consumption requirements for healthy adults^40^.

Evidence also suggests that CKD patients can adapt their muscle protein metabolism to a restricted protein intake^41,42^. A study that examined a “usual” diet (1.1 g/kg/day protein), an LPD (0.55 g/kg/day), or a supplemented VLPD (0.45 g/kg/day plus 0.1 g/kg/day amino/keto acids ) showed that skeletal muscles of CKD patients can respond to an LPD or decreased nitrogen intake to minimize muscle nitrogen losses and preserve muscle mass through the combined effects of reduced protein degradation, unchanged protein synthesis, and overall increased efficiency of recycling protein metabolism (i.e., amino acids deriving from protein breakdown)^41^. Moreover, in comparing the impact of plant-based diets vs. animal-based diets on muscle protein synthesis, studies in healthy adults have revealed that soy protein promotes resting muscle protein synthesis at comparable levels (vs. whey protein) or greater levels (vs. casein protein)^43^ and improves bench press strength, squat/leg press strength, or lean body mass (vs. animal proteins)^44^.

Another common misconception is that all plant-based diets are LPDs. Notably, data from 71,751 participants in the Adventist Health Study-2 has shown that strict vegetarians (~71 g/day) had a similar amount of dietary protein as non-vegetarians (~75 g/d)^45^. Hence, when considering plant-based diets for CKD patients, those with stage 3–5 NDD-CKD should be directed towards diet with lower dietary protein intake (0.6–0.8 g/kg/day) such as the PLADO^46^ or PLAFOND diets^47^.

With respect to the quality of dietary protein, most animal and soy proteins are generally considered high quality or complete protein sources for humans and contain adequate levels of essential amino acids to support human growth^48^. Although individual plant proteins, with the exception of soy protein, have insufficient levels of one or more essential amino acids, meals with different plant protein sources provide complete proteins throughout the day^49^.

## Conclusion

In summary, LPDs and supplemented VLPDs combined with essential amino acids or keta acid analogs are a mainstay in the management of NDD-CKD patients as a means to reduce CKD progression while maintaining optimal nutritional status and avoiding PEW. Future studies are needed to investigate the safety, effectiveness, and implementation of plant-based LPDs in reducing the progression of CKD and its complications in the real world.
